# Knowledge, attitude and practice of tomato retailers towards hygiene and food safety in Harar and Dire Dawa, Ethiopia

**DOI:** 10.1016/j.foodcont.2022.109441

**Published:** 2023-03

**Authors:** Biruk Alemu Gemeda, Kebede Amenu, Sisay Girma, Delia Grace, Ramasamy Srinivasan, Ralph Roothaert, Theodore J.D. Knight-Jones

**Affiliations:** a International Livestock Research Institute (ILRI), P.O. Box 5689, Addis Ababa, Ethiopia; b College of Veterinary Medicine and Agriculture, Addis Ababa University, P.O. Box 34, Bishoftu, Ethiopia; c College of Veterinary Medicine, Haramaya University, P.O.Box 138, Dire Dawa, Ethiopia; d Natural Resources Institute, University of Greenwich, Central Avenue, Chatham Maritime, Kent, ME4 4TB, UK; e Animal and Human Health Program, International Livestock Research Institute, Box, 30709, Nairobi, Kenya; f Safe and Sustainable Value Chains Flagship Program, World Vegetable Center, Tainan, 74151, Taiwan; g World Vegetable Center, Eastern and Southern Africa, P.O.Box 10, Arusha, Tanzania

**Keywords:** Knowledge, Attitude, Practice, Retailers, Tomato, Food safety

## Abstract

In this study, we assessed knowledge, attitude, and practices (KAP) related to tomato hygiene and food safety, among tomato vendors in the Ethiopian cities of Harar and Dire Dawa. From a total of 1498 tomato retail market vendors identified in the two cities through vendor mapping exercises, 151 outlets were randomly selected for a cross-sectional KAP survey on tomato handling, marketing, loss due to damage, safety, and hygienic practice. Tomato vendors claimed that they knew about food safety and hygiene, and risks associated with raw tomatoes. We found considerable variation in food safety knowledge, barriers, and practices during handling and marketing. The major concern of tomato traders in terms of food safety for vegetables was contamination with dirt. Around 17% of street vendors did not know about the importance of water quality and cleanliness for food safety. About 20% of tomato traders washed tomatoes after they purchased them and 43% and 14% of respondents who practiced tomato washing revealed that they cannot get the quantity and quality of water needed, respectively. Tomatoes were displayed in direct sunlight in about 85% of stalls. About 37% of vendors said rodents were present at night and could contact surfaces tomatoes are displayed on. For about 40% of outlets one or more flies were seen to be present on a third to two-thirds of their tomatoes. Overall, 40% of respondents reported they do not have adequate toilet facilities and 20% of those that use a toilet do not have water for washing hands after. The study identified areas that should be targeted by interventions aiming to improve food safety in this setting, however, without improvements in basic infrastructure to provide the pre-requisites for food safety the impact of small-scale food safety interventions may be limited.

## Highlights

• Basic infrastructures are lacking in the dominant informal food sector in Ethiopia.
• We found 40% of tomato retailers did not have adequate toilet facilities.
• Almost half did not have adequate access to clean water.
• Stalls sell live chickens in proximity to vegetables, which are often eaten raw.
• Food waste through damage was a problem for a quarter of retailers.

## 1 Introduction

Foodborne diseases (FBD) are responsible for cause major public health, economic and social burdens across the world. The impacts of FBD are amplified by increasing population mobility and the globalization of food supply (Faour-Klingbeil, Todd, & E, 2019; Pires et al., 2021).

Asia and sub-Saharan Africa (SSA) have the highest incidence of FBD, as well as the highest rate of deaths (2.5 times global average) due to FBDs and the greatest loss of Disability Adjusted Life Years (DALYs). Urbanization is rapidly accelerating in Africa and more than 80% of this food is marketed through informal value chains (VCs) (Grace et al., 2014). Most previous investment has focused on exports and formal markets whereas most of the FBD health burden falls on consumers of food from domestic informal markets (Grace, 2015).

A study estimated that the productivity losses alone attributed to unsafe food related illnesses in Africa are $20 billion in 2016, and the cost of treating these illnesses is an additional $3.5 billion (Jaffee et al., 2019). FBD is a considerable impediment to smallholder farmers who wish to sell in high value domestic and export markets that demand greater food safety assurances (Grace et al., 2018).

FBDs can disproportionately affect urban consumers because of the greater complexity of food VC supplying these markets, the long distances between their rural production and urban consumption centres, and the greater number of middlemen and food handling nodes (Grace, 2015). Although we know that fresh foods sold in informal markets account for a sizable proportion of the FBD burden there is limited information at country level on the priority hazards, the health risks, economic costs, or options for management. Moreover, there is a marked discrepancy between what consumers, VC actors, and policy makers are most concerned about and what is actually causing the FBD burden in terms of foods and hazards (Grace et al., 2018). These knowledge gaps make it difficult for policy makers to prioritize and manage food safety.

Outbreaks of FBD often result from problems with food hygiene during food handling (Wahida Salleh et al., 2017). Proper hygiene is essential for all food handlers. FBDs relating to the consumption of fruits and vegetables is widely reported (Goodburn & Wallace, 2013; “Microbiological Safety Evaluations and Recommendations on Fresh Produce,” 1999; Ssemanda et al., 2017; Van Boxstael et al., 2013). Many FBDs incidents have been attributed to fresh fruits and vegetables that became contaminated with microbes from on-farm or post-harvest water used for irrigation or cleaning (Arah et al., 2016).

Recently, food safety issues in fresh produce supply chain with particular reference to sub-Saharan Africa have been documented (Aworh, 2021). Central wholesale markes have an important position in fresh produce supply chain in SSA. These markets typically lack physical facilities and infrastructure including electricity and potable water from municipal sources and waste disposal facilities. There is critical need for improved packaging systems, refrigerated transport and cold chains for perishable produce in SSA for better produce quality and food safety (Aworh, 2021).

However, very little information is available regarding knowledge, attitude and practice of fruit and vegetable retailers in markets and food service facility towards hygiene and safety. There is a need to conduct research to better understand awareness, concerns and food safety challenges experienced by vendors who are key actors in value chains. Such information is imperative for the development and implementation of food safety intervention strategies.

There is a need to better control FBD in Ethiopia (Azanaw et al., 2019; Mendedo et al., 2017). Although animal sourced foods play a major role in FBD, vegetables are also responsible for a sizeable burden of FBD (Gazu et al., 2021), with pathogen contamination occurring through irrigation water, manure and cross-contamination from various sources from production to consumption. The risk is greater for vegetables commonly consumed raw such as leafy vegetables and tomatoes. Studies elsewhere showed fresh fruits and vegetables are increasingly linked to food-borne illnesses, outbreaks and product recalls (Jay-Russell, 2013).

In Ethiopia the vegetable subsector has a vital role in human nutrition and health, farm income generation, poverty alleviation and foreign currency earnings through export and direct foreign investment (Emana et al., 2017). Tomatoes are an essential part of diets in Ethiopia and are consumed in large quantities in many traditional dishes such as soups, sauces, stews and salads (Brasesco et al., 2019). However, although there are FBD risks associated with tomatoes, especially when eaten raw, there is little quantitative evidence available about KAP regarding food safety of retailers. These data are needed to understand and quantify the risks associated with tomato consumption and to guide strategies on how to improve food safety.

To fill this gap, within the “Pull-Push Project - Urban Food Markets in Africa – incentivizing food safety using a pull-push approach”, we assessed KAP about tomato hygiene and safety, among vendors involved in tomato retail and wholesale marketing in Harar and Dire Dawa, both large cities in Eastern Ethiopia.

## 2 Materials and methods

### 2.1 Study area

**Fig. 1:**
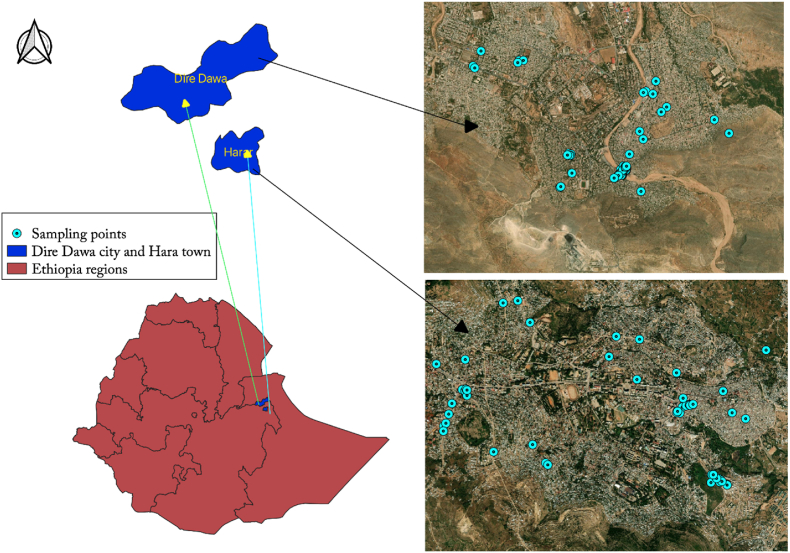
Map of Ethiopia showing regions and sampling sites.

This study was conducted in two major cities of Eastern Ethiopia, Dire Dawa (Ethiopia's 7th most populous city, population = 466,000, geographic coordinate: 9.6009° N, 41.8501° E) and Harar (Ethiopia's 16th most populous city, population = 246,000, geographic coordinate: 9.3126° N, 42.1227° E), located 515 KM and 510 km east of Addis Ababa (Fig. 1). The elevation of Dire Dawa is 1204 m above sea level and that of Harar is 1917 m (CSA, 2017/2018). Both cities are multi-ethnic: with Oromo, Amhara, Harari, Gurage, Somali, Tigray, and Argobba in Harar; and Oromo, Somali, Amhara, and Gurage in Dire Dawa.

A food safety project conducted tomato value chain assessments and mapping to give a general overview of tomato production, processing, retail and consumption practices with particular emphasis on food safety (Amenu et al., 2021). It found that in both cities vegetables are widely marketed and consumed but they are no major areas of local horticulture production, with much of the produce coming from central Ethiopia. Onion, tomato, potato, and lettuce were reported to be the first, second, third and fourth most highly consumed vegetables in the areas. Among vegetables, tomato was mainly eaten raw by washing with water and sometimes cooked with other vegetables, and consumption of raw lettuce was also common.

There are various types of vegetable retailers. Some retailers are located next to wholesalers in the main market of the cities, and these have stalls and supply tomatoes in bulk using wooden crates and cardboard boxes. The second type has fixed stalls in the vicinity of the main food market. They mostly buy tomatoes from wholesalers and large retailers (first type). The third type comprises street vendors not located in or by a market. They are the smallest, simplest and most informal type of outlet; however, most consumers obtain their food from them. They sell tomatoes in public spaces particularly roadsides offering easy access to customers. They account for a significant proportion of the vegetable traders that supply to consumers in both cities. Formal shops are the last type of retail outlets where a wide range of goods including tomatoes are offered for sale in a fixed, formal establishments.

### 2.2 Survey of tomato retailers

#### 2.2.1 Study design and sampling

A cross-sectional quantitative KAP survey was conducted in February 2021 in the two cities. First, a sampling frame of 1498 retailers was prepared based on a tomato market GPS mapping study in December 2020 to January 2021 (Amenu et al., 2021; Gemeda et al., 2021). The sampling frame was constructed by exhaustive mapping by walking along all streets in the two cities and was assumed to represent more than three-quarters of the potential vegetable market outlets. From the identified outlets, 151 were randomly selected without stratification, providing a representative sample (Fig. 1, Table 1).

**Table 1:** Number of tomato outlets in sampling frame and sampled by type and city.

	Sampling frame			Sampled		
	Dire Dawa	Harar	Total	Dire Dawa	Harar	Total
Retailers in market	215 (13.8%)	171 (28.7%)	386 (26%)	19 (21%)	17 (27%)	36 (24%)
Stalls in the vicinity of market	419 (46.5%)	273 (45.8%)	692 (46%)	32 (35%)	27 (44%)	59 (39%)
Street vendors not in market	214 (23.7%)	74 (12.4%)	288 (19%)	32 (35%)	6 (10%)	38 (25%)
Formal shop	54 (6%)	78 (13.1%)	132 (9%)	6 (7%)	12 (19%)	18 (12%)
**Total**	902 (100%)	596 (100%)	1498 (100%)	89 (100%)	62 (100%)	151 (100%)
	**60%**	**40%**	**100%**	**59%**	**41%**	**100%**

Unlike other stall types, those within a particular market were not individually identified in the sampling frame. To select the appropriate number of stalls from a particular market transect walks were performed. For example, if we needed five retailers in a given market, we located one retailer around the center and the remaining in four equally spaced-out locations at nearly equal distance from the central point to the edge of the market. If a selected vendor would not participate or was not selling tomatoes at the time (an eligibility requirement) we selected the adjacent suitable market outlet, or if outside the market the next outlet from the reserve list in the randomization selection process.

#### 2.2.2 KAP survey instrument and data collection

The survey instrument was developed by food safety experts and peer reviewed from a multidisciplinary team from International Livestock Research Center (ILRI) and World Vegetable Center. The data collection was pretested and set up in Open Data Kit (ODK) on mobile tablet devices. It contained the following sections: 1) background and demographic information of respondents, 2) tomato handling practices, and 3) food safety knowledge, attitude, and practice. In addition to questions, observations related to tomato handling, marketing, loss due to damage, safety and hygienic practice were entered. The instrument also covered for opinions on potential food safety interventions, perceptions and potential sources of food safety risks and barriers to behavior change around tomato safety. Enumerators were recruited and trained on how to administer the instrument. Thereafter, the instrument was piloted with 12 respondents and necessary modifications were made after that. Overall, each interview took about 50 min to complete.

Ethics approval was obtained from the Institutional Research Ethics Committee of the International Livestock Research Institute (ILRI-IREC2019-36). The study was explained, and consent was received from each respondent.

### 2.3 Data management and analysis

The collected data were downloaded from the ODK server in excel format and cleaned. Descriptive analysis was done using STATA version 16. Data were presented using tables (frequencies and percentages), and box plots, violin plots and bar charts. Categorical data were analyzed using Chi-square tests and continuous data using t-tests and one-way ANOVA considering means and standard deviations comparing different locations and type of outlet.

## 3 Results

### 3.1 Survey of tomato traders/retailers

#### 3.1.1 Demography of respondents and tomato market characteristics

##### 3.1.1.1 Vendor characteristics

The respondents' demographic and market characteristics are summarized in Table 2. Most were female adults with an average age of 33 years, and most were owners of the tomato outlet (only 7.3% were hired workers/employees). The majority of tomato traders sold several types of vegetables (96%) and a few also sold fruit (2.7%) (Table 2).

**Table 2:** General characteristics of participants and tomato markets.

Categorical variable	Category	Harar (n = 62)		Dire Dawa (n = 89)		Total (n = 151)	
		n	%	n	%	n	%
Participant gender	Female	44	71%	87	97.8%	131	86.7%
	Male	18	29%	2	2.2%	20	13.3%
Vendor type	Retailer in market	17	27.4%	19	21.3%	36	23.8%
	Stall in vicinity of market	27	43.5%	32	35.9%	59	39.1%
	Street vendors/Roadside stall not in or by market	6	9.7%	32	35.9%	38	25.2%
	Formal shop	12	19.4%	6	6.7%	18	11.9%
Participant role	Owner	55	88.7%	85	95.5%	140	92.7%
	Employee	7	11.3%	4	4.5%	11	7.3%
Animals roaming in the market	Yes	35	92.1%	45	88.2%	80	89.9%
	No	3	7.9%	6	11.8%	9	10.1%
Animals type roaming	Cattle	27	43.5%	11	12.4%	38	25.2%
	Sheep	31	0.5%	43	48.3%	74	49%
	Goat	26	41.9%	45	50.6%	71	47%
	Chicken	4	6.4%	7	7.8%	11	7.3%
	Dog	7	11.3%	11	12.3%	18	11.9%
	Cat	1	1.6%	0	0	1	0.6%
	Horse	0	0	4	4.5%	4	2.6%
Live animals sold at the outlet	yes	10	16.1%	19	21.3%	29	19.2%
	No	52	83.9%	70	78.7%	122	80.8%
Animals type sold	Chicken	9	14.5%	19	21.3%	28	18.5%
	Other (sheep and goat)	1	1.6%	3	3.4%	4	2.6%
Other items sold	Vegetable	57	91.9%	88	98.8%	145	96%
	Fruit	2	3.2%	2	2.2%	4	2.7%

The daily quantity of tomatoes sold varied with the type of vendors and location. The estimated average minimum and maximum quantities of tomato sold per day for retailers in market were 64.4 kg and 254.7 kg in Harar and 43.6 kg and 120.3 kg in Dire Dawa, respectively.

Tomato traders bought tomatoes from different sources, which varied according to the type of vendor (p = 0.05). About 58% of retailers obtained tomatoes from wholesalers while most street vendors received tomatoes from local middlemen (Table 3).

**Table 3:** Source of tomatoes for different tomato outlets.

	Wholesale		Producer		Small market outlet		Local middleman		Middleman (>100 km away)		
**Market Outlet type**	n	%	n	%	n	%	n	%	n	%	p-value
Retailer in market (n = 36)	22	61.1	1	2.8	0		6	16.7	7	19.4	0.007
Stall in vicinity of market (n = 59)	22	37.3	0	0	5	8.5	22	37.3	3	5.1	0.36
Street vendors/Roadside stall not in or by market (n = 38)	13	34.2	0	0	4	10.5	22	57.9	0	0	0.278
Formal shop (n = 18)	13	72.2	0	0	1	5.6	5	27.8	0	0	0.003

##### 3.1.1.2 Presence of animals

**Fig. 2:**
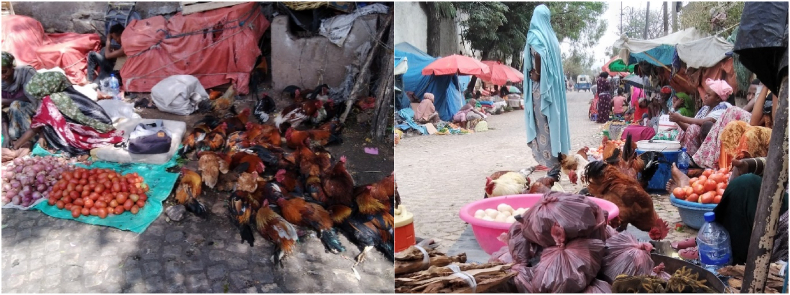
Tomato and live chicken are often sold in close proximity, Arategna, Harar.

Most of the respondents reported the presence of free-roaming animals in the marketplace. Sheep and goats were the major animal species roaming at the marketplace grazing on market waste. Most of the respondents did not sell live animals but 18.5% of outlets sold live chickens, often kept in proximity to the vegetables being sold (Fig. 2).

#### 3.1.2 Knowledge and perception of tomato traders regarding tomato safety and hygiene

In this study, 59% of participants had heard of consumers becoming sick from eating raw tomatoes such as found in salads. About 96% said that cleanliness and hygiene were important for customers when choosing where they buy vegetables. However, the major concerns of tomato traders in terms of food safety for vegetables was contamination with dirt rather than germs (Table 4).

**Table 4:** Knowledge and attitude of tomato traders regarding tomato safety and hygiene in Harar (n = 62) and Dire Dawa (n = 89).

Question asked	Location	Yes		No		Don't know	
		N	%	N	%	N	%
Do you ever hear about health problems from eating uncooked tomatoes such as in salads?	Harar	27	43.6%	35	56.5%		
	Dire Dawa	32	35.9%	57	64%		
	**Total**	**59**	**39.1%**	**92**	**60.9%**		
Is cleanliness and hygiene important to your customers when choosing where to buy vegetables?	Harar	60	96.8%	2	3.2%		
	Dire Dawa	85	95.5%	4	4.5%		
	**Total**	**145**	**96%**	**6**	**4%**		
Is water quality and cleanliness important for food safety?	Harar	53	85.5%	3	4.8%	6	9.7%
	Dire Dawa	79	88.8%	0	0	10	11.2%
	**Total**	**132**	**87.4%**	**3**	**1.9%**	**16**	**10.6%**
Is the temperature the food is kept at important for food safety?	Harar	55	88.7%	1	1.6%	6	9.7%
	Dire Dawa	86	96.6%	0	0	3	3.4%
	**Total**	**141**	**93.4%**	**1**	**0.6%**	**9**	**6%**
Is food waste through damage and spoilage of tomatoes a problem for you?	Harar	21	33.9%	41	66.1%		
	Dire Dawa	19	21.4%	70	78.7%		
	**Total**	**40**	**26.5%**	**111**	**73.5%**		
		**Food contaminated with dirt**		**Food contaminated with germs**		**Food contaminated with chemical**	
		N	%	N	**%**	N	%
What are you most concerned about in terms of food safety of vegetables?	**Harar**	39	62.9%	22	35.5%		
	**Dire Dawa**	63	70.8%	24	26.9%	2	2%
	**Total**	**102**	**67.5%**	**46**	**30.5**	**2**	**1.3%**

Around 87% of respondents in our study knew that water quality and cleanliness were important for food safety (Table 4). Compared to other outlet types a higher proportion of street vendors (17%) did not know about the importance of water quality and cleanliness for food safety.

Only 7% of the study participants were unaware that the temperature food was kept at was important for food safety (Table 4). Over a fifth of respondents (22.5%) mentioned that food waste through damage and spoilage of tomatoes was a problem for them.

Retailers were aware of different measures which could reduce tomato waste (Table 5). Sorting produce according to level of damage was practiced by most actors in all outlet types. Stalls in the vicinity of markets focused on better storage and packaging. Just under 10% of retailers mentioned better tomato management during production, such as pesticide application, as being important for reducing damage and wastage. Most actors (70%) did not recognize washing as a measure to reduce tomato food wastage. They said tomatoes do not “like” washing (sic.) and become damaged more quickly after washing.

**Table 5:** Measures to reduce tomato waste mentioned as important by different tomato market outlets.

Market outlet type	Sorting		Washing		Limiting transport distance		Better storage		Better packaging		Better management at production (e.g., pesticide application)	
	n	%	n	%	n	%	n	%	n	%	n	%
Retailer in market (n = 36)	33	91.7	8	22.2	8	22.2	16	44.4	21	58.3	3	8.3
Stall in vicinity of market (n = 59)	59	100	23	38.9	21	35.6	36	61	42	71.2	0	0
Street vendors (n = 38)	37	97.4	8	21.1	10	26.3	13	34.2	13	34.2	0	0
Formal shop (n = 18)	16	88.9	6	33.3	4	22.2	11	61.1	4	22.2	0	0
**Total**	**145**	**96%**	**45**	**29.8**	**43**	**28.3**	**76**	**50.3**	**80**	**52.9**	**3**	**8.3**
**p-value**	**0.078**		**0.182**		**0.459**		**0.046**		**0.000**		**0.004**	

#### 3.1.3 Critical control points and practice of safety and hygiene of tomato

Potential determinants of tomato safety and hygiene and possible re-contamination areas were documented for each relevant practice to help identify possible focus areas for tomato food safety interventions.

##### 3.1.3.1 Tomato handling

Most respondents (69%), particularly retailers in markets (75%), obtained tomatoes packed in wooden crates, and 66% of respondents considered this the best container for tomatoes. Plastic bags were considered the worst container for tomatoes and were used by 2% of respondents.

##### 3.1.3.2 Sorting

Tomato traders sort tomatoes according to quality during different market activities. Around 27% of respondents often sort tomatoes when they sell them, while 41% sort whenever tomatoes are handled. Only 5% do not practice sorting when they sell tomato. A higher proportion of respondents also reported the practice of sorting during transport and storage to avoid loss due to cross contamination of damaged and heathy tomatoes (Table 6).

**Table 6:** Handling practices reported by vendors when they sell, transport and store tomatoes in Harar and Dire Dawa.

	Location	NA	Never	Rarely	Sometimes	Often	Always
Do you sort tomatoes according to quality when you sell them?	H (n = 40)	12.5%	7.5%	5%	12.5%	20%	42.5%
	D (n = 63)	6.4%	3.2%	4.8%	14.3%	31.8%	39.7%
	**Total (n = 103)**	**8.7%**	**4.9%**	**4.9%**	**13.6%**	**27.2%**	**40.8%**
Do you sort tomatoes according to quality when you transport them.?	H (n = 40)	30%	12.5%	5%	10%	30%	12.5%
	D (n = 63)	30.2%	11.1%	6.4%	15.9%	20.6%	15.9%
	**Total (n = 103)**	**30.1%**	**11.7%**	**5.8%**	**13.6%**	**24.3%**	**14.6%**
Do you sort tomatoes according to quality when stored?	H (n = 40)	15%	10%	5%	15%	37.5%	17.5%
	D (n = 63)	9.5%	7.9%	6.4%	15.9%	42.9%	17.5%
	**Total (n = 103)**	**11.7%**	**8.7%**	**5.8%**	**15.5%**	**40.8%**	**17.5%**
Do tomatoes become damaged when they are being taken or transported to storage?	H (n = 62)	53.2%	27.4%	19.4%	0	0	0
	D (n = 89)	49.4%	26.9%	17.9%	2.3%	2.3%	1.1%
	**Total (n = 151)**	**50.9%**	**27.2%**	**18.5%**	**1.3%**	**1.3%**	**0.7%**
In storage are tomatoes exposed to flies?	H (n = 62)	3.2%	41.9%	35.5%	12.9%	6.5%	0
	D (n = 89)	3.4%	30.3%	28.1%	30.3%	6.7%	1.1%
	**Total (n = 151)**	**3.3%**	**35.1%**	**31.1%**	**23.2%**	**6.6%**	**0.7%**
In storage are they exposed to animals/rodents?	H (n = 62)	9.7%	53.2%	17.7%	12.9%	6.5%	0
	D (n = 89)	5.6%	50.6%	15.7%	24.7%	2.3%	1.1%
	**Total (n = 151)**	**7.3%**	**51.7%**	**16.6%**	**19.9%**	**3.9$**	**0.7%**

##### 3.1.3.3 Storage

Most of the traders (81%) usually store tomatoes at the stall or in a room near the stall at night; 9% take tomatoes to their home and some reported other mechanisms like storing tomatoes in a friend's home or in a common secured storage place (10%). Traders usually transport/carry tomatoes on foot to the storage place (86%). The average time it takes to get tomato to storage location for street vendors is 8 min and the rest market outlets takes less. The minimum and maximum number of days between when outlets receive a batch of tomatoes and when they sell it, for intact tomatoes is longer in formal shops compared to other outlets. The average minimum and maximum days between when outlets receive a batch of tomatoes and when they sell them, for intact tomatoes for retailers in markets in Harar is 3.5 and 6 days, respectively, and 5 and 9 days in formal shop outlets. In Dire Dawa, the average minimum and maximum days between when outlets receive a batch of tomatoes and when they sell them, for intact tomatoes for retailers in market is 1 and 3 days, respectively, whereas 4 and 6 days in formal shop outlets.

##### 3.1.3.4 Exposure to flies and rodents

About 35% and 52% of respondents self-reported that tomatoes were not exposed to flies and rodents when in storage while the rest had a varying degree of exposure (Table 6).

##### 3.1.3.5 Tomato marketing

Tomatoes were displayed in direct sunlight at most stalls in the vicinity of tomato markets (85%) and street vendors (87%) visited, more commonly than seen in retailers in markets (42%) and formal shops (28%): this was more prevalent in Dire Dawa (79%) than in Harar (53%). Tomatoes were exposed to sunlight for a long period of time in the absence of an umbrella or any kind of shade.

In this study, only about 20% of tomato traders practiced washing tomatoes after they purchased them but the majority of these (97%) used just water and never use detergent or soap to wash fresh tomatoes. About 15% of the traders regularly washed the tomatoes on display (Table 7). Most traders (81%) use dry cloth or other dry item to remove dust from the tomatoes on display during the day, while 40.4% use wet cloth to wash/clean the tomatoes.

**Table 7:** Practices relevant to tomato safety and hygiene.

Practice questions	Location	Yes		No		Sometimes	
		N	%	N	%	N	%
Do you wash the tomatoes after you purchase them? *	Harar	17	27.4%	42	67.7%	3	4.8%
	Dire Dawa	3	3.4%	80	89.9%	6	6.7%
	**Total**	20	13.3%	122	80.8%	9	5.9%
Of those that do above - Do you wash with just water?	Harar	20	100%	0	0		
	Dire Dawa	8	88.9%	1	11.1%		
	**Total**	28	96.6%	1	3.4%		
Do you wash the tomatoes when on display? *	Harar	16	25.8%	46	74.2%		
	Dire Dawa	7	7.9%	82	92.1%		
	**Total**	23	15.2%	128	84.7%		
Of those that do above - Do you wash with just water?	Harar	16	100%	0	0		
	Dire Dawa	7	100%	0	0		
	**Total**	23	100%	0	0		
Of those that wash tomatoes after purchase - Can you get the quantity of water that you need?	Harar	13	68.4%	6	31.6%		
	Dire Dawa	3	33.3%	6	66.7%		
	**Total**	16	57.1%	12	42.9%		
Of those that wash tomatoes after purchase - Can you get the quality of water that you need? *	Harar	18	94.7%	1	5.3%		
	Dire Dawa	6	66.7%	3	33.3%		
	**Total**	24	85.7%	4	14.3%		
Do you use a wet cloth to wash/clean the vegetables on display during the day?	Harar	25	40.3%	37	59.7%		
	Dire Dawa	36	40.5%	53	59.6%		
	**Total**	61	40.4%	90	59.6%		
Do you change this washcloth during the day? *	Harar	6	24%	19	76%		
	Dire Dawa	23	63.9%	13	36.1%		
	**Total**	29	47.5%	32	52.5%		
Do you use a dry cloth or other dry item to remove dust from the vegetables on display during the day?	Harar	48	77.4%	14	22.6%		
	Dire Dawa	74	83.2%	15	16.9%		
	**Total**	122	80.8%	29	19.2%		
Are toilet facilities adequate?	Harar	40	64.5%	22	35.5%		
	Dire Dawa	50	56.2%	39	43.8%		
	**Total**	90	59.6%	61	40.4%		
Is water available to wash hands after using the toilet?	Harar	49	79.1%	13	20.9%		
	Dire Dawa	72	80.9%	17	19.1%		
	**Total**	121	80.1%	30	19.9%		

**Fig. 3:**
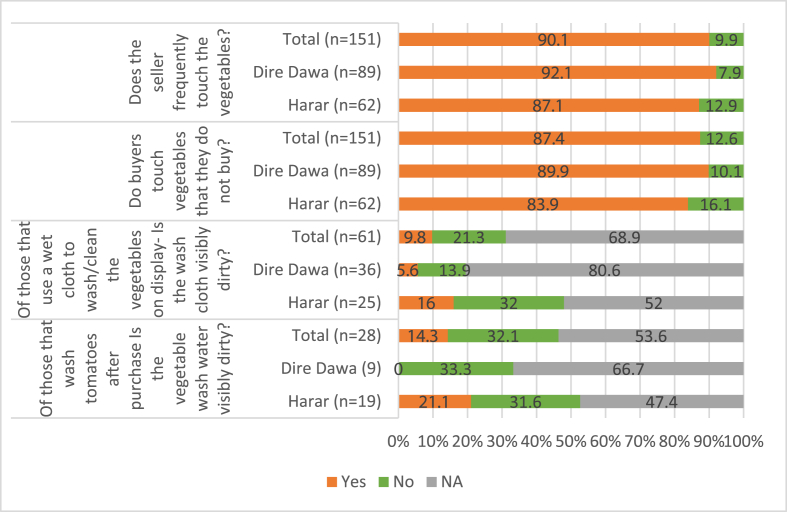
Enumerator observation of practices relevant to tomato safety and hygiene in percentages (NA-not applicable to evaluate/not seen during the enumerator's visit).

Customers frequently handled or touched vegetables for sale that they do not buy in 87% of outlets and vendors frequently handled vegetables for sale in 90% of outlets (Fig. 3). About 37% of vendors said rodents come at night and contact surfaces tomatoes are displayed on.

For about 40% of outlets one or more flies were seen on a third to two-thirds of tomatoes. About 55% of street vendors visited had one or more flies compared with 22% of formal shops. The fly occurrence was significantly higher in Dire Dawa (50%) than Harar (24%) (p = 0.005).

##### 3.1.3.6 Market infrastructure and facilities

**Fig. 4:**
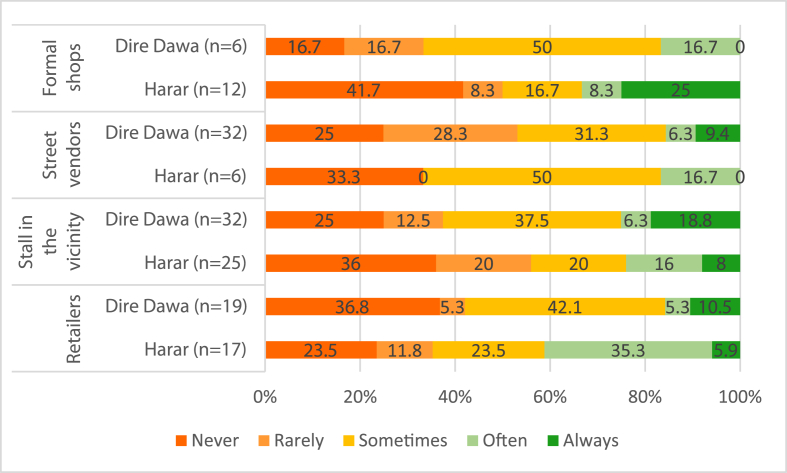
Percentage of respondents use soap or sanitizer during hand washing after going to the toilet by outlet type and location.

About 40% of respondents reported they do not have adequate toilet facilities and 20% of those that use a toilet do not have water for washing hands after. About 30% of respondents who wash their hands after going to the toilet never use soap or sanitizer (Fig. 4).

**Fig. 5:**
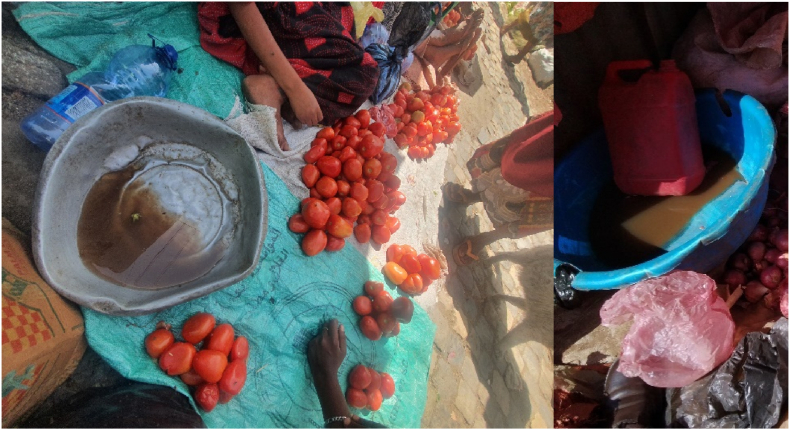
Tomatoes washed with dirty water in Shewaber, Harar.

Around 43% and 14% of respondents who washed tomatoes said that they cannot get the quantity and quality of water needed, respectively (Table 8) and 14% of the wash water is visibly dirty (Fig. 5) according to enumerator observation.

**Table 8:** Major reasons for damage to tomatoes reported for different tomato market outlet types.

	Soft tomatoes		During Transport		Pests		Kept at high temperature		Kept too long	
**Market Outlet type**	n	%	n	%	n	%	n	%	n	%
Retailer in market (n = 36)	17	47.2	30	83.3	13	36.1	14	38.9	15	41.7
Stall in vicinity of market (n = 59)	40	67.8	45	76.3	22	37.3	27	45.8	43	59
Street vendors/Roadside stall not in or by market (n = 38)	12	31.6	22	57.9	9	23.7	30	78.9	25	65.8
Formal shop (n = 18)	12	66.7	15	83.3	4	22.2	10	55.6	11	61.1
p-value		0.03		0.051		0.385		0.002		0.023
**Study location**										
Harar (n = 62)	31	50	48	77.4	19	30.6	29	46.7	32	51.6
Dire Dawa (n = 89)	50	56.2	64	71.9	29	32.6	52	58.4	62	69.6
Total (n = 151)	81	53.6	112	74.2	48	31.8	81	53.6	94	62.3
P-value		0.454		0.447		0.801		0.158		0.02

Waste disposal facilities were not observed in tomato outlets and about 98% of respondents revealed that the wastewater from the outlets were disposed of on the ground.

##### 3.1.3.7 Amount of damaged tomatoes and source of damage

**Fig. 6:**
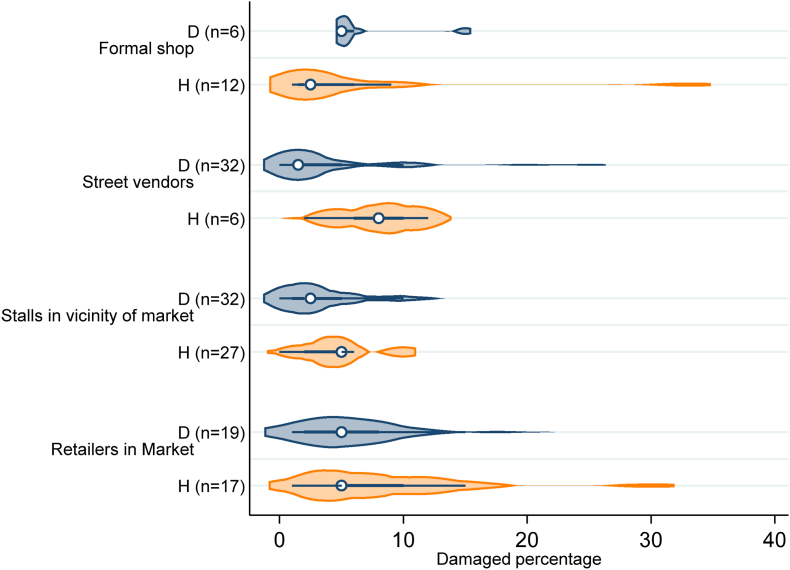
Percentage of damaged tomatoes for each tomato retail outlet in Harar and Dire Dawa.

In this study, a damaged tomato was defined as one where the surface is clearly broken, and thus cannot be sold at the highest price. The result revealed the proportion of damaged tomatoes varied for different outlet types (p = 0.043) and location (Fig. 6). The mean percentage of tomatoes sold that are damaged was highest for retailers in the market (7%) and lowest for stalls in the vicinity of markets (4%). The proportion of damaged tomato was also higher in Harar (6%) than in Dire Dawa (5%) (p = 0.08).

The main perceived cause of tomato damage was being kept for long periods of time and high storage temperatures in Dire Dawa, while damage during transport was frequently mentioned in Harar. The reasons of damage varied significantly among outlet types and study location (Table 8).

## 4 Discussion

Mature and well-governed food value chains may be able to reduce FBD by requiring and incentivizing high standards along the value chain, with pre-requisite infrastructure, audits and appropriate verification of key steps and sometimes product testing. However, in developing countries the expansion of food value chains is happening without effective governance, know-how or facilities, resulting in uncontrolled food safety risks (Grace, 2015). Government oversight of food systems is often inadequate. As a result, individual value chain actors and consumers are left with the responsibility for recognizing food safety risks and ensuring food hygiene when they purchase and handle foods.

To reliably produce food that is safe requires good practices by all the different participants within the food system, starting with producers and transporters, moving through food processors and retailers, down to consumers with oversight from governments (Hosking et al., 2020).

In this study, the shortage of basic market infrastructure and services, such as potable water supplies and unacceptable market environment clearly indicate a lack of oversight. The presence of free roaming animals at the marketplace and live chickens sold very close to tomatoes and other vegetables will act as a source for microbial contamination for foodborne pathogens through direct deposition of fecal material on tomatoes or indirectly with faecal-borne pathogens becoming deposited on the tomatoes via the environment, fomites, and handling. Food-producing animals are the major reservoirs for many foodborne pathogens such as *Campylobacter* species, non-Typhi serotypes of *Salmonella enterica*, Shiga toxin-producing strains of *Escherichia coli*, and *Listeria monocytogenes* (Heredia & García, 2018).

The study also found that a substantial proportion of respondents do not have adequate toilet facilities and those that use a toilet do not have water and soap for washing hands. Human faecal-oral route of transmission is another key pathway for foodborne pathogen spread, including via contaminated food after unhygienic handling. Besides, vendors and customers frequently handled vegetables for sale in the majority of outlets. Tomatoes are vulnerable to contamination with, and subsequent growth of, pathogenic micro-organisms, which may be passed from personnel in contact with the produce, and the unhygienic conditions which may be present in retailing (frequent handling of produce by vendors and consumers) (FAO, 2018). Providing easily accessible toilet and hand washing facilities for tomato traders to use is critical for preventing contamination of tomatoes. As is minimal handling of the produce.

Keeping food at the proper temperature is one of the most important things a food handler can do to prevent growth of microorganisms that cause foodborne illness (WHO, 2006). Although only a few of the study participants were unaware that the temperature the food kept at is important for food safety, most normally store tomatoes at the stall without proper infrastructure to maintain appropriate storage temperatures facilitating microbial survival and growth (N.B. ambient monthly average daytime temperatures in the study sites ranges from 24 °C to 31 °C in 2021), and wastewater from outlets was disposed on the ground in the market with inadequate waste disposal facilities. Such liquid waste should be discharged into the sewer or the drain.

The study also showed that food hygiene perceptions and vendor practices (for example sorting, washing, protection from direct sunlight, prevention of flies and rodents) at chosen points, were also inadequate. A limitation of the study was reliance on recall of self-reported practices and the possibility of either inaccurate reporting or social desirability bias; by combining self-reports with direct observation, we were able to address this bias to some extent.

Most of the participants of the study had heard of cases of people becoming sick after eating raw tomatoes such as in salads and said that cleanliness and hygiene were important for customers when choosing where to buy vegetables. Consumers and key-informants during the previous value chain analysis (Amenu et al., 2021) had repeatedly mentioned their concerns about the use of chemicals in vegetable production. However, the major concerns of tomato traders in terms of vegetable food safety were contamination with dirt rather than germs and chemicals, perhaps as it is more visible and easier to comprehend and making the produce harder to sell. Similar findings were documented by Curtis (1988) who underlined the importance of recognizing the strong social dimensions of hygiene in developing countries. Dirt-avoidance was a desirable behavior long before the discovery of bacterial disease transmission (Curtis, 1988). Thus, public awareness should be provided showing that hygiene is not only about the removal of dirt but also avoidance hazards posed by certain germs.

Water quality is critical for maintaining the safety of the product (FAO and WHO, 2019). In our study, most of respondents apart from some street vendors knew this. But respondents revealed that they cannot get the quantity and quality of water needed, reflecting the need to increase access to clean water.

Although most retailers used wooden crates and considered this as the best container for tomatoes, routine practices like packing in wooden crates with rough and pointed edges, over-packing and stacking of non-uniform crates can cause mechanical damage to tomatoes (Hosking et al., 2020). The use of rigid crates made from polypropylene, high-density polyethylene or similar plastics is recommended for fresh fruits and vegetables in SSA (Aworh, 2021). Though evidence on the benefits of reusable plastic crates (RPCs) in Ethiopia is scarce, GAIN analysis indicates a 50–75% reduction in losses from using RPCs compared to wooden crates (Hosking et al., 2020) most likely by reducing mechanical damage.

Sorting, believed to minimize further tomato loss and potential cross-contamination (Arah et al., 2016), is the removal of rotten, damaged, or diseased fruits from the healthy and clean ones. The damaged or diseased fruits can produce ethylene in substantial amounts, which can affect the adjacent fruits causing them to over-ripen and become soft and prone to damage. In our study, although a higher proportion of vendors sorted tomatoes according to different qualities during transport, storage and at selling, effort is still needed to get these practices more widely and routinely implemented by street vendors. Once damaged tomatoes better support microbial survival and growth increasing FBD risks (Ogundipe et al., 2012).

Tomatoes were displayed in direct sunlight in most stalls and street vendors visited during the study. This reduces the shelf life of tomatoes, and the quality is compromised when exposed to high temperatures and high relative humidity (Arah et al., 2016). When retailing in open-air markets and roadside stalls, tomatoes on display should be shaded from sun protection (FAO, 2018).

Most actors did not recognize washing as a food safety measure or something that reduces tomato food wastage. They said tomatoes do not “like” washing, with the moisture causing softening and accelerated damage. However, many did regularly wash tomatoes without disinfectants or use wet cloth to clean the tomatoes, principally to make them visibly more appealing to customers. Water quality is a critical contributing factor for both food safety and product quality. Workneh et al. (2012) indicated that anolyte water dipping disinfection of tomatoes not only reduced the microbial loads on the fruits but also maintained superior quality of tomatoes during storage (Workneh et al., 2012). However, washing with dirty water has the potential to facilitate transmission of pathogens between vegetables. The water and the cloth used to wipe the fruit must be clean to prevent contaminating the fruit (FAO, 2018).

The finding also showed rodents come at night and contact surfaces tomatoes are displayed on. Tomatoes on display were also commonly contaminated with flies reflecting a lack of hygienic practices in many outlets (40%). Rodents can be reservoirs and vectors of a number of agents that cause disease in food animals and humans (e.g. *Leptospira* spp., *Salmonella* spp., *Campylobacter* spp., *Trichinella* spp., *Toxoplasma* spp.). Flies readily move between wastes and foods, transporting microorganisms with them as they go (Songe et al., 2016). Tomato traders should be aware of the need for pest control from a food safety perspective. Preferably, rodent and fly control should form an integral part of a total package of hygiene measures to prevent transfer of food-borne pathogens (Black et al., 2018; Meerburg & Kijlstra, 2007). However, without adequate infrastructure, such as clean water, cleanable surfaces and equipment, animal proof facilities improvements in food safety brought about by better food safety knowledge and practices may be restricted.

Food safety is not a stand-alone development objective but must be balanced with other concerns such as livelihoods, equity and nutrition (Grace, 2015). This study found that tomato retail was influenced by gender with women dominating. This is typical of informal markets in Africa (Grace et al., 2015). This highlights the importance of tomato retail to women's livelihoods and gender equity, and implies any intervention on retailers aimed at improving hygiene and food safety should target women. We also found that the informal sector remains the most important source of tomatoes and this should be reflected in efforts to improve food safety and reduce the massive foodborne disease burden experienced in Africa. However, although some practices in informal markets are risk amplifying (e.g., exposure to pests) others are risk mitigating (e.g., more rapid sale).

In conclusion, this study found knowledge variation and fundamental gaps in terms of good food safety practice amongst tomato vendors, resulting in poor hygiene and safety among tomato outlets in informal food value chains. The study also identified areas that should be targeted by interventions aiming to improve food safety in this setting.

The result highlights the need to educate vendors on food hygiene, and the need for basic equipment and infrastructure in order to provide safe food. Interventions could take the form of simple food safety packages including provision and promotion of plastic tomato crates; training on food safety and hygienic practices, clean environments and provision of basic infrastructure and services, such as potable water supplies, facilities and waste management.

It is likely that these findings are applicable elsewhere across sub-Saharan Africa where such informal markets dominate the food sector. Given the massive burden of FBD in these settings it is imperative that prompt action is taken.

## Funding

This study was funded by the 10.13039/100000865Bill & Melinda Gates Foundation, UK Government Foreign, Commonwealth & Development Office (FCDO) - UK Aid from the United Kingdom government (INV-008430-OPP1195588) and the CGIAR Research Program on Agriculture for Nutrition and Health. The funder played no role in the design or conclusion of the study.

## CRediT authorship contribution statement

**Biruk Alemu Gemeda:** followed up and monitored data collection, Formal analysis, Writing – original draft. **Kebede Amenu:** Conceptualization, Methodology, followed up and monitored data collection. **Sisay Girma:** followed up and monitored data collection. **Delia Grace:** Conceptualization, Methodology. **Ramasamy Srinivasan:** Conceptualization, Methodology. **Ralph Roothaert:** Conceptualization, Methodology. **Theodore J.D. Knight-Jones:** Conceptualization, Methodology, All authors read, commented and approved the final manuscript.

## Declaration of competing interest

The authors declare that they have no known competing financial interests or personal relationships that could have appeared to influence the work reported in this paper.

## Data Availability

Data will be made available on request.

## References

[bib1] Amenu K., Bedasa M., Wamile M., Worku H., Kasim K., Taha M., Mego L., Dinede G., Ssemanda J.N., Grace D., Roesel K., Roothaert R., Srinivasan R., Knight-Jones T. (2021).

[bib2] Arah I.K., Ahorbo G.K., Anku E.K., Kumah E.K., Amaglo H. (2016). Postharvest handling practices and treatment methods for tomato handlers in developing countries: A mini review. Advances in Agriculture.

[bib3] Aworh O.C. (2021). Food safety issues in fresh produce supply chain with particular reference to sub-Saharan Africa. Food Control.

[bib4] Azanaw J., Gebrehiwot M., Dagne H. (2019). Factors associated with food safety practices among food handlers: Facility-based cross-sectional study. BMC Research Notes.

[bib5] Black E.P., Hinrichs G.J., Barcay S.J., Gardner D.B. (2018). Fruit flies as potential vectors of foodborne illness. Journal of Food Protection.

[bib6] Brasesco F., Asgedom D., Casari G. (2019).

[bib7] Curtis V. (1988).

[bib8] Emana B., Afari-Sefa V., Nenguwo N., Ayana A., Kebede D., Mohammed H. (2017). Characterization of pre- and postharvest losses of tomato supply chain in Ethiopia. Agriculture & Food Security.

[bib9] FAO (2018).

[bib10] Faour-Klingbeil D., Todd C.D., E (2019). Prevention and control of foodborne diseases in middle-east north african countries: Review of national control systems. International Journal of Environmental Research and Public Health.

[bib11] FAO and WHO (2019). Safety and quality of water used in food production and processing: Meeting report. Microbiological Risk Assessment series.

[bib12] Gazu L., Maximiano F., Ulrich P., Guadu T., Grace D., Alonso S., Mutua F., Roesel K., Lindahl J., Amenu K., Ilboudo G., Dione M., Knight-Jones T. (2021).

[bib13] Gemeda B.A., Amenu K., Mego L., Dione M., Ilboudo G., Lallogo V.R., Girma S., Kasim K., Taha M., Knight-Jones T. (2021). Presentation at a webinar for the “Urban food markets in Africa” project.

[bib14] Goodburn C., Wallace C.A. (2013). The microbiological efficacy of decontamination methodologies for fresh produce: A review. Food Control.

[bib15] Grace D. (2015). Food safety in developing countries: An Overview.

[bib16] Grace D., Alonso S., Lulietto M., Kebede A., Lindahl J., Mader R., Madukeh V. (2018).

[bib17] Grace D., Kang’ethe E., Bonfoh B., Roesel K., Makita K. (2014). Presented at the 4th annual leverhulme centre for integrative research on agriculture and health (LCIRAH) conference, london, UK, 3-4 june 2014.

[bib18] Grace D., Roesel K., Kang’ethe E., Bonfoh B., Theis S. (2015).

[bib19] Heredia N., García S. (2018). Animals as sources of food-borne pathogens: A review. Animal Nutrition.

[bib20] Hosking S., Amin M., Zena Z., Yalch T., Nordhagen S. (2020). Business models for reducing post-harvest loss of fresh vegetables.

[bib21] Jaffee S., Henson S., Unnevehr L., Grace D., Cassou E. (2019). https://openknowledge.worldbank.org/handle/10986/30568.

[bib22] Jay-Russell M.T. (2013). What is the risk from wild animals in food-borne pathogen contamination of plants?. CAB Reviews: Perspectives in Agriculture, Veterinary Science, Nutrition and Natural Resources.

[bib23] Meerburg B.G., Kijlstra A. (2007). Role of rodents in transmission ofSalmonella andCampylobacter. Journal of the Science of Food and Agriculture.

[bib24] Mendedo E.K., Berhane Y., Haile B.T. (2017). Factors associated with sanitary conditions of food and drinking establishments in Addis Ababa, Ethiopia: Cross-sectional study. Pan African Medical Journal.

[bib25] Microbiological safety evaluations and recommendations on fresh produce (1999). Food Control.

[bib26] Ogundipe F.O., Bamidele F.A., Adebayo-Oyetoro A.O., Ogundipe O.O., Tajudeen O.K. (2012). Incidence of bacteria with potential public health implications in raw Lycopersicon esculentum (tomato) sold in lagos state, Nigeria. Nigerian Food Journal.

[bib27] Pires S.M., Desta B.N., Mughini-Gras L., Mmbaga B.T., Fayemi O.E., Salvador E.M., Gobena T., Majowicz S.E., Hald T., Hoejskov P.S., Minato Y., Devleesschauwer B. (2021). Burden of foodborne diseases: Think global, act local. Current Opinion in Food Science.

[bib28] Salleh W., Nizam Lani M., Zawiah Wan Abdullah W., Zainazor Tuan Chilek T., Zaiton Hassan (2017). A review on incidences of foodborne diseases and interventions for a better national food safety system in Malaysia. Malaysian Applied Biology.

[bib29] Songe M., Hang’ombe B., Knight-Jones T., Grace D. (2016). Antimicrobial resistant enteropathogenic Escherichia coli and Salmonella spp. in houseflies infesting fish in food markets in Zambia. International Journal of Environmental Research and Public Health.

[bib30] Ssemanda J.N., Reij M., Bagabe M.C., Muvunyi C.M., Joosten H., Zwietering M.H. (2017). Indicator microorganisms in fresh vegetables from “farm to fork” in Rwanda. Food Control.

[bib31] Van Boxstael S., Habib I., Jacxsens L., De Vocht M., Baert L., Van De Perre E., Rajkovic A., Lopez-Galvez F., Sampers I., Spanoghe P., De Meulenaer B., Uyttendaele M. (2013). Food safety issues in fresh produce: Bacterial pathogens, viruses and pesticide residues indicated as major concerns by stakeholders in the fresh produce chain. Food Control.

[bib32] WHO (2006). Five keys to safer food manual. World Health Organization.. DEPARTMENT OF FOOD SAFETY, ZOONOSES AND FOODBORNE DISEASES.

[bib33] Workneh T.S., Osthoff G., Steyn M. (2012). Effects of preharvest treatment, disinfections, packaging and storage environment on quality of tomato. Journal of Food Science & Technology.

